# Priority Strategy of Intracellular Ca^2+^ Homeostasis in Skeletal Muscle Fibers during the Multiple Stresses of Hibernation

**DOI:** 10.3390/cells9010042

**Published:** 2019-12-22

**Authors:** Jie Zhang, Xiaoyu Li, Fazeela Ismail, Shenhui Xu, Zhe Wang, Xin Peng, Chenxi Yang, Hui Chang, Huiping Wang, Yunfang Gao

**Affiliations:** 1 Key Laboratory of Resource Biology and Biotechnology in Western China, College of Life Sciences, Northwest University, Ministry of Education, Xi’an 710069, China; zhangjie1@stumail.nwu.edu.cn (J.Z.); rjfazila123@gmail.com (F.I.); xushenhui@stumail.nwu.edu.cn (S.X.); wangzhe754778887@126.com (Z.W.); e1084875064@163.com (X.P.); wanghp@nwu.edu.cn (H.W.); 2 Shaanxi Key Laboratory for Animal Conservation, Northwest University, Xi’an 710069, China; 3 Human Functional Genomics Laboratory, Northwest University, Xi’an 710069, China; lixiaoyualian@gmail.com; 4 College of Biological Science and Engineering, North Minzu University, Yinchuan 750021, China; rain300861@163.com

**Keywords:** calcium homeostasis, hibernation, mitochondria, sarcoplasmic reticulum, skeletal muscle

## Abstract

Intracellular calcium (Ca^2+^) homeostasis plays a vital role in the preservation of skeletal muscle. In view of the well-maintained skeletal muscle found in Daurian ground squirrels (*Spermophilus dauricus*) during hibernation, we hypothesized that hibernators possess unique strategies of intracellular Ca^2+^ homeostasis. Here, cytoplasmic, sarcoplasmic reticulum (SR), and mitochondrial Ca^2+^ levels, as well as the potential Ca^2+^ regulatory mechanisms, were investigated in skeletal muscle fibers of Daurian ground squirrels at different stages of hibernation. The results showed that cytoplasmic Ca^2+^ levels increased in the skeletal muscle fibers during late torpor (LT) and inter-bout arousal (IBA), and partially recovered when the animals re-entered torpor (early torpor, ET). Furthermore, compared with levels in the summer active or pre-hibernation state, the activity and protein expression levels of six major Ca^2+^ channels/proteins were up-regulated during hibernation, including the store-operated Ca^2+^ entry (SOCE), ryanodine receptor 1 (RyR1), leucine zipper-EF-hand containing transmembrane protein 1 (LETM1), SR Ca^2+^ ATPase 1 (SERCA1), mitochondrial calcium uniporter complex (MCU complex), and calmodulin (CALM). Among these, the increased extracellular Ca^2+^ influx mediated by SOCE, SR Ca^2+^ release mediated by RyR1, and mitochondrial Ca^2+^ extrusion mediated by LETM1 may be triggers for the periodic elevation in cytoplasmic Ca^2+^ levels observed during hibernation. Furthermore, the increased SR Ca^2+^ uptake through SERCA1, mitochondrial Ca^2+^ uptake induced by MCU, and elevated free Ca^2+^ binding capacity mediated by CALM may be vital strategies in hibernating ground squirrels to attenuate cytoplasmic Ca^2+^ levels and restore Ca^2+^ homeostasis during hibernation. Compared with that in LT or IBA, the decreased extracellular Ca^2+^ influx mediated by SOCE and elevated mitochondrial Ca^2+^ uptake induced by MCU may be important mechanisms for the partial cytoplasmic Ca^2+^ recovery in ET. Overall, under extreme conditions, hibernating ground squirrels still possess the ability to maintain intracellular Ca^2+^ homeostasis.

## 1. Introduction

The maintenance of cytoplasmic calcium (Ca^2+^) homeostasis is important for the preservation of a normal structure and function of skeletal muscle fibers. Skeletal muscle inactivity can trigger Ca^2+^ homeostasis disturbance, often characterized by cytoplasmic Ca^2+^ overload [1]. A direct consequence of this overload is the activation of calpain system-mediated protein degradation [2]. In addition, an increased cytoplasmic Ca^2+^ concentration can promote cell apoptosis [3]. Increased protein degradation and cell apoptosis are both involved in skeletal muscle loss.

Hibernation is a unique survival strategy exhibited by various mammals in order to cope with adverse environments in winter, during which hibernators not only face the challenge of prolonged skeletal muscle inactivity, but also deal with other stresses, including hypoxia, fasting, and repeated ischemia-reperfusion during the torpor-arousal cycle. However, various studies have reported that skeletal muscle is well-maintained in hibernators during hibernation [4,5]. Therefore, hibernators can be considered typical anti-atrophy models, with their unique skeletal muscle preservation mechanism undoubtedly an attractive and valuable research topic.

Previous findings from our laboratory showed that, under adverse conditions over several months of hibernation, the cytoplasmic Ca^2+^ concentration in skeletal muscle fibers of Daurian ground squirrels increased transiently during inter-bout arousal, partially recovered after re-entering torpor, and almost recovered to pre-hibernation levels in the post-hibernation stage, thus exhibiting good Ca^2+^ homeostasis during the entire hibernation cycle [6]. During long-term hibernation, the torpor-arousal cycle likely plays an important role in protecting skeletal muscle from atrophy by avoiding or alleviating persistent and excessive cytoplasmic Ca^2+^ overload-induced protein degradation. Therefore, exploring the potential mechanisms involved in Ca^2+^ homeostasis during hibernation could help reveal the mechanisms against disuse-induced skeletal muscle atrophy of hibernators. To date, however, only one study (from our lab) has reported on sarcoplasmic reticulum Ca^2+^ pump (SERCA) expression in skeletal muscles during hibernation [7]. As such, the regulatory mechanisms involved in intracellular Ca^2+^ homeostasis in skeletal muscle fibers are far from having been clarified.

The level of intracellular Ca^2+^ is closely related to the expression level and activity of Ca^2+^ transport proteins or channels located in the plasma membrane and intracellular Ca^2+^ storage membrane (mainly sarcoplasmic reticulum (SR) and mitochondria), as well as intracellular Ca^2+^ binding proteins. Increased extracellular Ca^2+^ influx and intracellular Ca^2+^ storage/release (especially in the SR) both contribute to an increase in the intracellular Ca^2+^ concentration. Store-operated Ca^2+^ entry (SOCE) is the most important channel transporting extracellular Ca^2+^ into the cytosol. Stromal interaction molecule-1 (STIM1) located in the endoplasmic reticulum (ER) and Orai1 (also known as calcium-release-activated calcium-modulator, CRACM1) located in the cell membrane are two essential components required for SOCE [8,9,10]. With external stimulation, Ca^2+^ is released from the STIM1 EF-hand domain, which triggers the aggregation and movement of STIM1 to ER/plasma membrane (PM) binding sites, as well as the Orai1 aggregation of STIM1, and leads to the activation of SOCE and Ca^2+^ influx [11,12,13,14]. The ryanodine receptor (RyR) is a major SR Ca^2+^ release channel. Specifically, when sensing cell membrane depolarization, exterior membrane L-type calcium channels (surface membrane and T tubules) and dihydropyridine receptors (DHPR) combine to activate RyR, resulting in substantial SR Ca^2+^ release [15,16]. The RyR family is comprised of three isoforms (i.e., RyR1–3), with RyR1 exclusively expressed and particularly enriched in skeletal muscle [17]. Leucine zipper-EF-hand-containing transmembrane protein 1 (LETM1) is a Ca^2+^-H^+^ exchanger located in the mitochondrial membrane. When the mitochondrial Ca^2+^ concentration is high, LETM1 will extrude excess Ca^2+^ from the mitochondria into the cytoplasm [18]. Therefore, LETM1 is another possible contributor to elevated cytoplasmic Ca^2+^ levels.

In contrast to the above mechanisms, however, the increase in Ca^2+^ efflux, intracellular Ca^2+^ uptake of the Ca^2+^ pool, and binding capacity of free Ca^2+^ binding protein in the cytoplasm all effectively decrease cytoplasmic Ca^2+^. Plasma membrane Ca^2+^ ATPase (PMCA) can eject Ca^2+^ from the cytosol into the external medium, thereby attenuating the cytoplasmic Ca^2+^ concentration. PMCA3 is the major isoform expressed in skeletal muscle [19]. As a primary active transporter located in the SR membrane, SR/ER Ca^2+^ ATPase (SERCA) can decrease cytoplasmic Ca^2+^ levels by pumping Ca^2+^ from the cytosol into the SR, which is one of the key factors attenuating cytoplasmic Ca^2+^ overload in skeletal muscle fibers [20]. The mitochondrial calcium uniporter (MCU) complex is considered a major channel for the transportation of Ca^2+^ into mitochondria [21]. Mitochondrial calcium uptake 1 and 2 (MICU1 and 2) are two regulatory subunits of MCU [22]. When the Ca^2+^ concentration in the intermembrane space is low, the heterodimers of MICU1 and MICU2 block the MCU channel and inhibit the entry of Ca^2+^ into the mitochondria. In contrast, when the Ca^2+^ level is high upon stimulation, the binding of Ca^2+^ to the MICU protein elicits a conformational change, resulting in the opening of the channel and the transportation of Ca^2+^ into the mitochondria [21,23]. Calmodulin (CALM), a Ca^2+^ binding protein located in the cytoplasm, can directly reduce the concentration of cytoplasmic free Ca^2+^ by combining with four Ca^2+^ ions [24]. Overall, Ca^2+^ uptake channels, extrusion mechanisms, and free Ca^2+^ binding proteins all contribute to intracellular Ca^2+^ homeostasis.

What, then, is the role of Ca^2+^ channels in Ca^2+^ fluctuations during the torpor-arousal cycle? To answer this question, we investigated the cytoplasmic, SR, and mitochondrial Ca^2+^ levels in the plantaris (PL, calf muscle) and adductor magnus (AM, thigh muscle) muscles of Daurian ground squirrels during different hibernation states (i.e., summer active, pre-hibernation, late torpor (entering a new bout after more than 5 d), inter-bout arousal (arousing spontaneously for less than 12 h), early torpor (entering a new bout for less than 48 h), and post-hibernation). Furthermore, a comprehensive and time-course investigation was carried out to explore the roles of the above major Ca^2+^ transport proteins/channels, including SOCE, RyR1, LETM1, PMCA3, SERCA1, and MCU, as well as the major Ca^2+^ binding protein CALM, in the fluctuations of Ca^2+^ concentration throughout hibernation.

## 2. Materials and Methods

### 2.1. Animals and Groups

**Figure 1:**
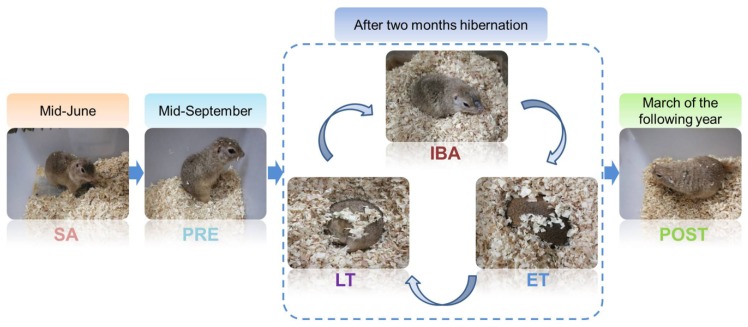
Representative images and sampling time of Daurian ground squirrels in different periods. SA, summer active; PRE, pre-hibernation; LT, late torpor; IBA, inter-bout arousal; ET, early torpor; POST, post-hibernation.

All animal procedures and care and handling protocols were in accordance with the approval granted by the Laboratory Animal Care Committee of the China Ministry of Health (approval No. MH-55). The Daurian ground squirrels used in the experimental procedures were captured from the Weinan region, Shaanxi Province, China. Upon return to the laboratory, all squirrels were maintained in an animal room under a temperature range of 18–25 °C and modified daily light conditions (coincident with local sunrise and sunset). After one month of adaptation, the adult individuals were weight-matched and divided into six groups (n = 6–8): (i) Summer active group (SA): samples were collected in mid-June; (ii) pre-hibernation group (PRE): samples were collected in mid-September; (iii) late torpor group (LT): after two months hibernation, animals entered into a new hibernation bout and were in continuous torpor for at least 5 d, with a stable body temperature (Tb) of 5–8 °C; (iv) inter-bout arousal group (IBA): after two months hibernation, animals entered into a new hibernation bout and were fully aroused, with the Tb returned to 34–37 °C for less than 12 h; (v) early torpor group (ET): after two months hibernation, animals entered into a new hibernation bout, with Tb maintained at 5–8 °C for less than 24 h; (vi) post-hibernation group in spring (POST): animals awaking from hibernation and maintaining a Tb of 36–38 °C for more than 3 d in March of the following year. Animals in the SA and PRE groups were maintained in an environment with a natural light:dark photoperiod until sacrifice. When the ground squirrels gradually entered torpor in early November, the animals were transferred to a 4–6 °C dark hibernaculum. Due to observations occurring twice a day under weak light, these animals were housed under a 2:22 light-dark cycle. The Tb of animals was measured using a visual thermometer with thermal imaging (Fluke, VT04, Everett, Washington, DC, USA). The different states of the animals used here are shown in Figure 1.

### 2.2. Muscle Sample Collection and Preparation

Muscle sample collection was carried out at 9:00 am for the SA, PRE, LT, and POST groups. Due to the specific sampling procedures in the IBA and ET groups, it could not be guaranteed that sample collection in these two groups always occurred at 9:00 am. Animals were anesthetized with sodium pentobarbital (90 mg/kg). The two distinct skeletal muscles (PL and AM) were carefully isolated and surgically removed. Subsequently, left leg muscles were treated with embedding medium for frozen section cutting and staining and right leg muscles were used for all other experiments. Following surgical intrusion, the squirrels were euthanized via sodium pentobarbital overdose injection.

### 2.3. Skeletal Muscle Fiber Cross-Sectional Area (CSA) Determination

As described previously [25], immunofluorescence and confocal analyses were used to measure the muscle fiber cross-sectional area (CSA) in frozen sections. Briefly, 10-μm thick frozen cross-sections were cut from the muscle mid-belly at −20 °C with a CM1850 cryostat (Leica, Wetzlar, Germany) and stored at −80 °C until further staining. After fixing in 4% paraformaldehyde for 30 min, slices were permeabilized in 0.1% Triton X-100 for 30 min, blocked with 1% bovine serum albumin (BSA) in phosphate-buffered saline (PBS) at room temperature for 60 min, and then incubated at 4 °C overnight with an anti-laminin antibody (1:500, Boster, BA1761-1, Wuhan, China) to visualize muscle fiber CSA. Subsequently, after washing them three times with PBS (10 min/time), the sections were incubated at 37 °C for 2 h with a 647-labeled IgG secondary antibody (1:400, Thermo Fisher Scientific, A-21235, Eugene, OR, USA). Finally, the slices were treated with anti-fade mounting medium (Life Technologies, 1427588, Eugene, OR, USA). Images were visualized via confocal laser scanning microscopy (Olympus, FV1000, Tokyo, Japan) at a 40× objective magnification. Image-Pro Plus 6.0 was used to measure muscle fiber CSA. In detail, eight images were captured from each sample, and the CSA of all complete muscle fibers (about 30) within each picture was then analyzed. Therefore, the CSA of ~250 muscle fibers per skeletal muscle sample was determined.

### 2.4. Single Muscle Fiber Isolation

We anaesthetized the squirrels with 90 mg/kg sodium pentobarbital, after which muscle samples (including the tendons) were carefully removed from the neighboring tissues and sarcolemma, ensuring that the blood and nerve supply remained intact. The muscle samples were subsequently separated into two full-length strips along the longitudinal axis using a pair of tweezers. The muscle strips, which were obtained from the same middle region, were then washed with 20 mL of PBS (137 mM sodium chloride, 2.7 mM potassium chloride, 4.3 mM disodium chloride, 1.4 mM monopotassium phosphate, pH 7.4) and digested with 3 mL of enzymatic digestion solution containing 0.35% collagenase I (Sigma-Aldrich, C0130-1G, Saint Quentin Fallavier, France), followed by orbital shaker incubation at 37 °C for 2 h and saturation with 95% O_2_ and 5% CO_2_ to ensure complete digestion of the muscle fiber samples. Finally, the digestion solution was removed with a PBS rinse and the muscle samples were added to Dulbecco’s modified Eagle’s medium (DMEM) (Hyclone, AC10221937, Pittsburgh, PA, USA) containing 10% fetal bovine serum (FBS) (Everygreen, 11011-8611, Hangzhou, China), 25 µM of N-benzyl-p-toluenesulfonamide (BTS) (TCI, B3082, Shanghai, China), and 0.1 M HEPES (Guoan, H0082, Xi’an, China), and were carefully stirred with a pipette. The digested single muscle fiber samples were finally plated on culture chamber slides and viewed via inverted microscopy (Olympus, IX2-ILL100, Tokyo, Japan).

### 2.5. Measurement of Cytoplasmic Ca^2+^

We used fluo-3-acetoxymethylester (Fluo-3/AM) (Invitrogen, Carlsbad, CA, USA), which demonstrates increased fluorescence upon Ca^2+^ binding, to determine cytoplasmic free Ca^2+^. Briefly, after washing the samples three times with fresh PBS, dye (5 mM Fluo-3/AM) was slowly added along the sides of the single muscle fibers, followed by incubation in the dark at 37 °C for 30 min. After incubation, the glass slide-mounted Fluo-3/AM-loaded fibers were washed with fresh PBS three times (20 s/time, 1-min process). The slide was quickly placed on the microscope stage, with the fibers focused in the bright field (20-s process) and scanned via laser confocal microscopy in combination with an Olympus FV10-ASW system (Tokyo, Japan) under 488-nm krypton/argon laser illumination, with fluorescence detected at 526 nm. According to their length, three to five pictures were captured at 10× objective magnification for each muscle fiber (10-s capture process for each picture). In consideration of the influence of muscle fiber size on the fluorescence intensity, the average fluorescence intensity (total fluorescence intensity/total area of selected region) was used to measure the Ca^2+^ levels. Specifically, the average fluorescence intensity of 10 different regions in each picture was measured using Olympus Fluoview v4.2 software. All pictures (3–5 pictures, depending on the muscle fiber length) of each fiber, with 10 muscle fibers per sample, were used for statistical analysis.

### 2.6. Measurement of Sarcoplasmic Reticulum Ca^2+^

We used magnesium-Fluo-4-acetoxymethylester (mag-Fluo-4/AM) (M14206, Thermo Fisher Scientific, Eugene, OR, USA), which demonstrates increased fluorescence upon Ca^2+^ binding, to indicate SR free Ca^2+^, as per Park et al. (2000) [26]. Briefly, after washing samples twice with fresh PBS, dye (5 mM mag-Fluo-4/AM) was slowly added along the sides of the single muscle fibers, followed by incubation in the dark at 37 °C for 30 min. After incubation, the glass slide-mounted mag-Fluo-4/AM-loaded fibers were washed with fresh PBS three times (20 s/time, 1-min process). The slide was then quickly placed on the microscope stage, with the fibers focused in the bright field (20-s process) and scanned via laser confocal microscopy in combination with an Olympus FV10-ASW system (Japan) under 488-nm krypton/argon laser illumination, with fluorescence detected at 526 nm. Analysis and statistical methods were similar to those used for the measurement of cytoplasmic Ca^2+^ mentioned above.

### 2.7. Measurement of Mitochondrial Ca^2+^

We used Rhod-2/AM (R1244, Thermo Fisher Scientific, USA), which demonstrates increased fluorescence upon Ca^2+^ binding in the mitochondria, to determine mitochondrial free Ca^2+^ [27]. Briefly, after washing samples twice with fresh PBS, dye (5 µM Rhod-2/AM) was slowly added along the sides of the single muscle fibers, followed by incubation in the dark at 37 °C for 30 min. After incubation, the glass slide-mounted Rhod-2/AM-loaded fibers were washed with fresh PBS three times (20 s/time, 1-min process). The slide was then quickly placed on the microscope stage, and the fibers were focused in the bright field (20-s process) and scanned via laser confocal microscopy in combination with an Olympus FV10-ASW system (Japan) under 594-nm krypton/argon laser illumination, with fluorescence detected at 618 nm. Analysis and statistical methods were similar to those used for the measurement of cytoplasmic Ca^2+^ mentioned above.

### 2.8. Total RNA Extraction and Quantitative Real-Time Polymerase Chain Reaction (RT-PCR)

As per Fu et al. (2016) [6] and in accordance with the manufacturer’s protocols, we extracted total RNA from the muscle samples using an RNAiso Plus kit (TaKaRa Biotechnology, 9109, Dalian, China). RNA quality was characterized using the OD260/OD280 ratio, after which selected samples (those exhibiting OD260/OD280 > 1.8) were reverse transcribed into cDNA using an appropriate reagent (TaKaRa Biotechnology, RR036A China) and stored (−20 °C) for the following analyses. Here, qRT-PCR was undertaken using a SYBR Premix Ex Taq II kit (TaKaRa Biotechnology, RR820A, China), following the protocols stated by the manufacturer. The resultant dissolution and amplification curves were observed and selected, with the α-tubulin reference gene and 2^−ΔΔct^ method then being applied to analyze the relative mRNA concentrations of STIM1, ORAI1, RyR1, LETM1, PMCA3, SERCA1, MCU, MICU1, MICU2, CALM, and α-tubulin. The primers used for the above genes (Sangon, Nanjing, China) are listed in Table 1.

### 2.9. Protein Extraction and Western Blotting Analysis

Muscle samples (~0.1 g) were weighed and fully homogenized with 1 mM RIPA Lysis Buffer (Heart, WB053A, Xi’an, China), 1% protease inhibitor cocktail (Heart, WB053B, Xi’an, China), and 1% phenylmethylsulfonyl fluoride (PMSF, Heart, WB053C, Xi’an, China). After 15 min of centrifugation at 4 °C and 15,000 rpm, the supernatants were removed and placed into new tubes, with soluble protein concentrations then detected using a Pierce^TM^ BCA Protein Quantitation kit (Thermo Fisher Scientific, 23227, USA). The supernatants were mixed with 1 × SDS loading buffer (100 mM Tris, 5% glycerol, 5% 2-β-mercaptoethanol, 4% SDS, and bromophenol blue, pH 6.8) at a 1:4 *v/v* ratio, followed by boiling and then storage at −20 °C for further analysis.

Western blotting procedures were as described by Zhang et al. (2017) [28]. In brief, we first separated the muscle protein extracts using SDS-PAGE on 10% Laemmli gels (acrylamide/bisacrylamide ratio of 37.5:1 for STIM1, ORAI1, LETM1, PMCA3, SERCA1, MCU, MICU1, CALM) and on 6% Laemmli gels (acrylamide/bisacrylamide ratio of 37.5:1 for RyR1), respectively. Following electrophoresis (for 60 min at 120 V), the proteins were electrically transferred to 0.45-μm pore polyvinylidene difluoride (PVDF) membranes (Millipore, IPVH00010, Merck kGaA, Darmstadt, Germany) using the Bio-Rad (1703930) semidry transfer apparatus (Hercules, CA, USA) at 15 V for 30–40 min. We then blocked the membranes at room temperature for 2 h using 5% skim milk in TBST (containing 10 mM Tris-HCl, 150 mM NaCl, 0.05% Tween-20, pH 7.6), followed by overnight incubation at 4 °C with primary STIM1 (1:1000, CST, 5668S, Danvers, MA, USA), ORAI1 (1:1000, Thermo, MA5-15776, Eugene, OR, USA), RyR1 (1:1000, CST, 8153S, USA), LETM1 (1:1000, CST, 14997S, USA), PMCA3 (1:1000, Abcam, ab3530, Cambridge, UK), SERCA1 (1:1000, CST, 4219S, USA), MCU (1:1000, CST, 14997S, USA), MICU1 (1:750, CST, 12524S, USA), and CALM (1:1000, CST, 4830S, USA) antibodies in TBST containing 0.1% BSA. The membranes where then washed three times with TBST (10 min/time), followed by 1.5-h incubation at room temperature with horseradish peroxidase (HRP)-conjugated anti-rabbit or anti-mouse secondary antibodies (Thermo Fisher Scientific, A27014, USA). The membranes were again washed with TBST (four times × 10 min), with the resulting immunoblots being visualized using enhanced chemiluminescence reagents (Thermo Fisher Scientific, NCI5079, USA), in accordance with the manufacturer’s instructions. Blot quantification was conducted using Image-Pro Plus 6.0 software. Total protein staining of the gel was used as the normalization control for all blots. In detail, as described previously, 0.5% 2,2,2-trichloroethanol (TCE) was first added to the gel [29,30,31]. After electrophoresis, the gel was irradiated on the UV platform of the electrophoresis gel imaging analysis system (G: box, GBOX Cambridge, UK) for 5 min, with the signal then being collected. As described previously [32,33], the original images captured with no gain were stored. After that, the fluorescence intensity of each lane (after removal of the background fluorescence intensity) was determined with Image-Pro Plus 6.0, with the internal reference being used to correct the fluorescence intensity of the target protein. Specificity detection of the complete SDS-PAGE lane for each antibody used in the present study is shown in Figure S1.

### 2.10. Co-Localization Analysis of ORAI1/STIM1

Briefly, 10-μm thick frozen cross-sections were cut from the muscle mid-belly at −20 °C with a CM1850 cryostat (Leica, Wetzlar, Germany). After fixing samples in 4% paraformaldehyde for 30 min, slices were permeabilized in 0.1% Triton X-100 for 30 min, blocked with 1% BSA in PBS at room temperature for 60 min, and then incubated at 4 °C overnight with an anti-ORAI1 antibody (1:50, Thermo, MA5-15776, USA). On the second day, after washing samples three times with PBS (10 min/time), the sections were incubated at 37 °C for 2 h with an Alexa Fluor FITC-conjugated secondary antibody (1:300, Thermo Fisher Scientific, Rockford, IL, USA). After again washing samples three times with PBS (10 min/time), the slices were incubated at 4 °C overnight with an anti-STIM1 antibody (1:300, CST, 5668S, USA). On the third day, after washing samples three times with PBS (10 min/time), the sections were incubated at 37 °C for 2 h with a 647-labeled IgG secondary antibody (1:200, Thermo Fisher Scientific, A-21235, USA). The slices were then washed three times with PBS (10 min/time) and dried and treated with anti-fade mounting medium (Life Technologies, 1427588, USA). Images were visualized and captured via confocal laser scanning microscopy (Olympus, FV1000, Japan) at a 40× objective magnification with krypton/argon laser illumination at 488 and 647 nm and captured at 526 and 665 nm. As previously described [34,35], the co-localization of ORAI1/STIM1 was calculated by Pearson’s correlation coefficients using Image-Pro Plus 6.0.

### 2.11. Statistical Analysis

Data are presented as means ± SEM. SPSS Statistics 17.0 was used for all statistical tests. Group differences were determined via one-way analysis of variance (ANOVA) with Fisher’s least significant difference (LSD) post hoc test. When no homogeneity was detected, ANOVA-Dunnett’s T3 method was applied. A value of *p* < 0.05 was considered statistically significant.

## 3. Results

### 3.1. Skeletal Muscle Mass and Single Muscle Fiber CSA of PL and AM during Different Hibernation Periods

**Figure 2:**
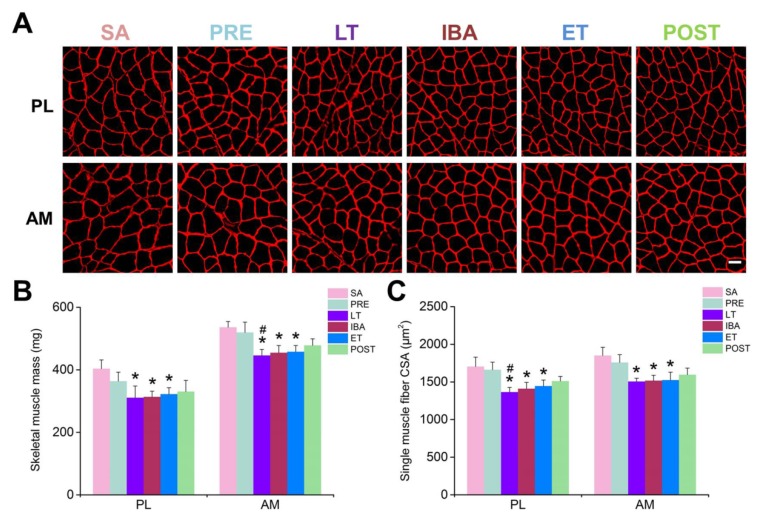
Changes in muscle mass and single muscle fiber CSA in PL and AM muscles during different periods. (**A**) Representative fluorescence images of single muscle fiber CSA in PL and AM muscles. 400× magnification, scale bar = 100 μm. (**B**) Histogram depicting muscle mass of PL and AM muscles during different periods. (**C**) Histogram depicting single muscle fiber CSA in PL and AM muscles during different periods. CSA, cross-sectional area; PL, plantaris; AM, adductor magnus. SA, summer active group; PRE, pre-hibernation group; LT, late torpor group; IBA, inter-bout arousal group; ET, early torpor group; POST, post-hibernation group. Values are means ± SEM, n = 6–8. * *p* < 0.05 compared with SA; # *p* < 0.05 compared with PRE.

Changes in skeletal muscle morphology were observed by analyzing the muscle mass (MM) (Figure 2B) and muscle fiber CSA (Figure 2A,C) in different groups. The results showed that, compared with the SA group, slight decreases in muscle mass and single muscle fiber CSA (15–20%) were observed during hibernation.

### 3.2. Cytoplasmic Ca^2+^ Level in Single Skeletal Muscle Fibers during Different Hibernation Periods

**Figure 3:**
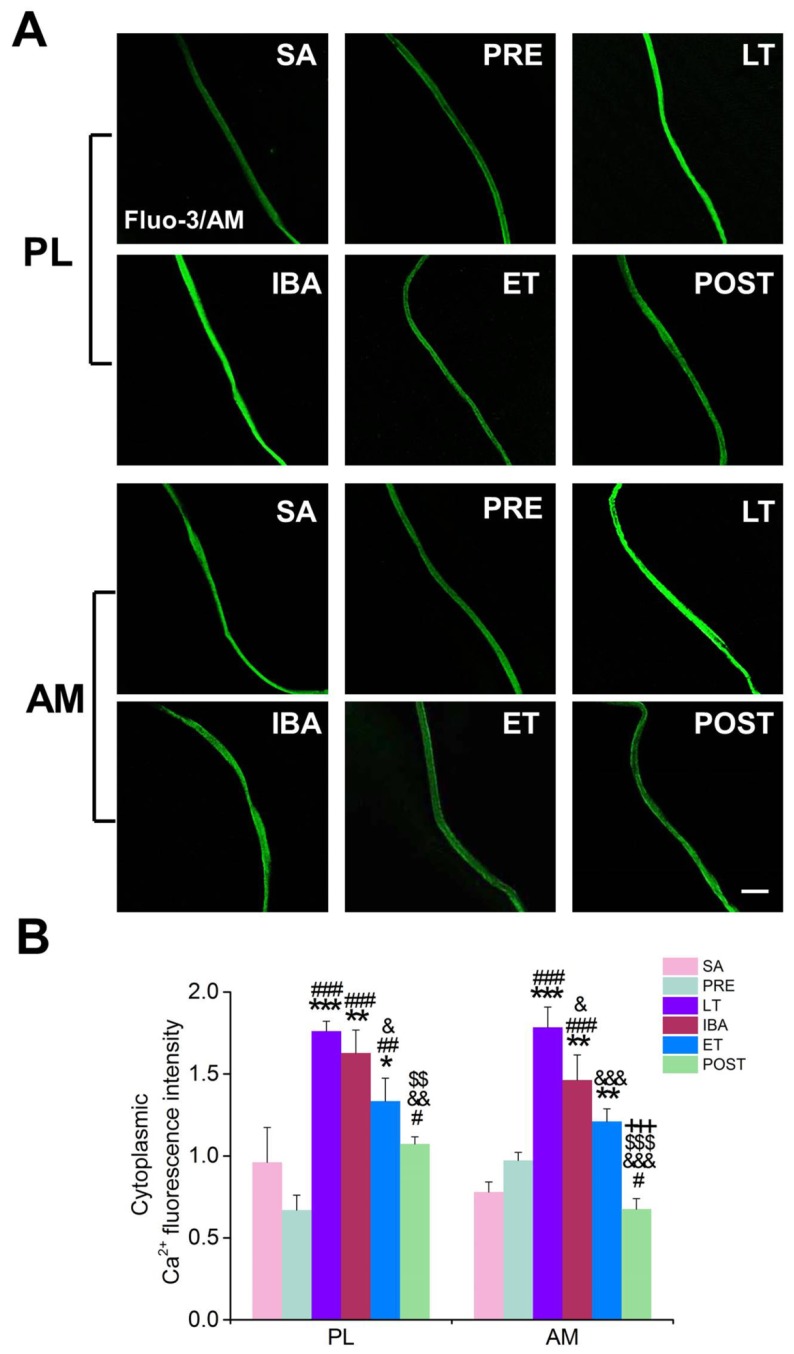
Changes in cytoplasmic Ca^2+^ concentration in PL and AM muscles during different periods. (**A**) Representative fluorescence images of single PL and AM muscle fibers. Scale bar = 100 μm. (**B**) Histogram depicting the cytoplasmic Ca^2+^ fluorescence intensity in PL and AM muscles during different periods. PL, plantaris; AM, adductor magnus. SA, summer active group; PRE, pre-hibernation group; LT, late torpor group; IBA, inter-bout arousal group; ET, early torpor group; POST, post-hibernation group. Values are means ± SEM, n = 6–8. * *p* < 0.05, ** *p* < 0.01, and *** *p* < 0.001 compared with SA; # *p* < 0.05, ## *p* < 0.01, and ### *p* < 0.001 compared with PRE; & *p* < 0.05, && *p* < 0.01, and &&& *p* < 0.001, compared with LT; $$ *p* < 0.01 and $$$ *p* < 0.001 compared with IBA; +++ *p* < 0.001 compared with ET.

In comparison with that in the SA and PRE groups, the cytoplasmic Ca^2+^ level in the single PL and AM muscle fibers increased significantly during hibernation, and almost recovered to SA levels post-hibernation. During the torpor-arousal cycle, the cytoplasmic Ca^2+^ level partially recovered when animals re-entered the torpor state. Compared with the results in the LT group, significant decreases in cytoplasmic Ca^2+^ levels were observed in the PL (24%) and AM muscles (32%) of the ET group (Figure 3).

### 3.3. SR Ca^2+^ Level in Single Skeletal Muscle Fibers during Different Hibernation Periods

**Figure 4:**
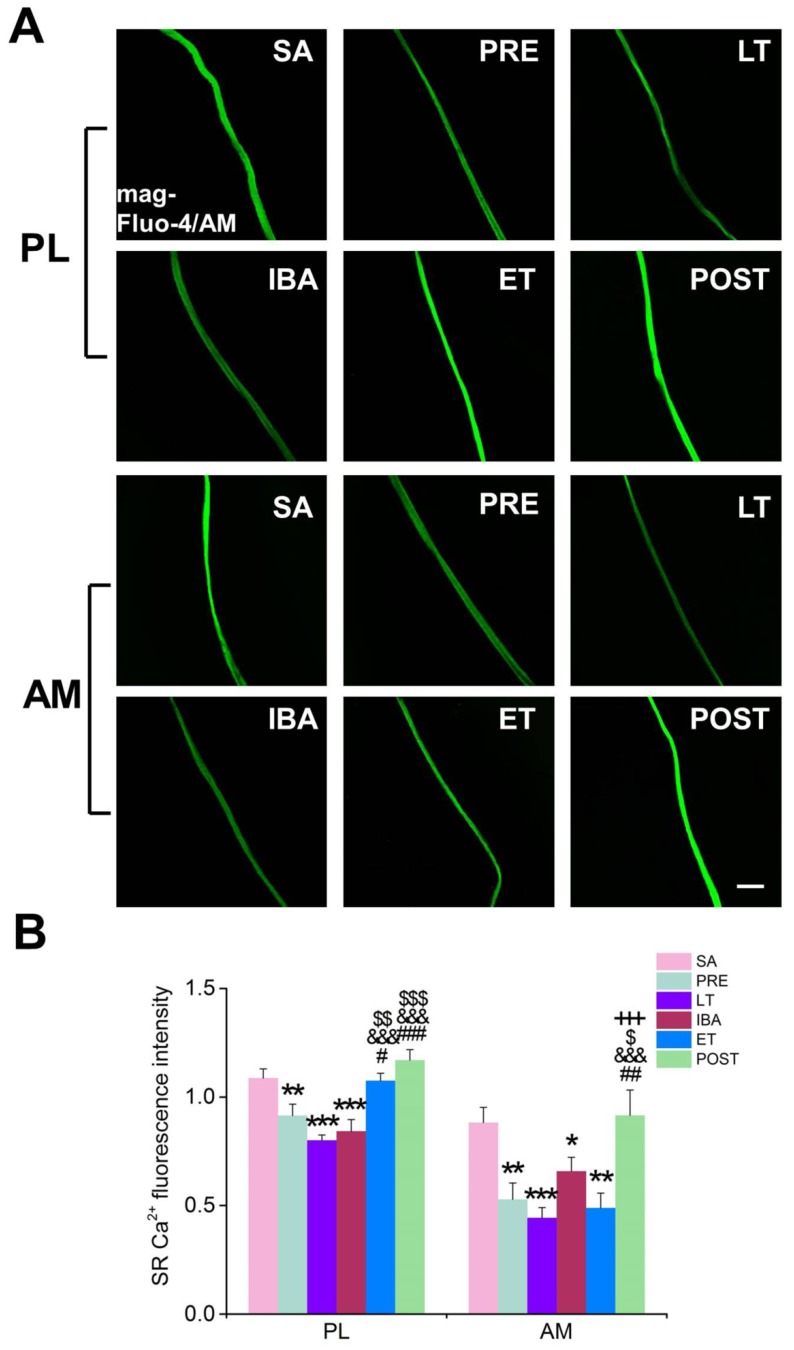
Changes in the SR Ca^2+^ concentration in PL and AM muscles during different periods. (**A**) Representative fluorescence images of single PL and AM muscle fibers. Scale bar = 100 μm. (**B**) Histogram depicting the cytoplasmic Ca^2+^ fluorescence intensity in PL and AM muscles during different periods. SR, sarcoplasmic reticulum; PL, plantaris; AM, adductor magnus. SA, summer active group; PRE, pre-hibernation group; LT, late torpor group; IBA, inter-bout arousal group; ET, early torpor group; POST, post-hibernation group. Values are means ± SEM, n = 6–8. * *p* < 0.05, ** *p* < 0.01, and *** *p* < 0.001 compared with SA; # *p* < 0.05, ## *p* < 0.01, and ### *p* < 0.001 compared with PRE; &&& *p* < 0.001 compared with LT; $ *p* < 0.05, $$ *p* < 0.01, and $$$ *p* < 0.001 compared with IBA; +++ *p* < 0.001 compared with ET.

In contrast to the results produced for cytoplasmic Ca^2+^, the SR Ca^2+^ level in single PL and AM muscle fibers decreased significantly during hibernation and almost recovered to SA levels post-hibernation (Figure 4). During the torpor-arousal cycle, compared with that in the LT and IBA groups, the SR Ca^2+^ level in the PL muscle was significantly elevated by 27–34% when animals re-entered the torpor state, indicating that the SR Ca^2+^ level partially recovered in the ET group.

### 3.4. Mitochondrial Ca^2+^ Level in Single Skeletal Muscle Fibers during Different Hibernation Periods

**Figure 5:**
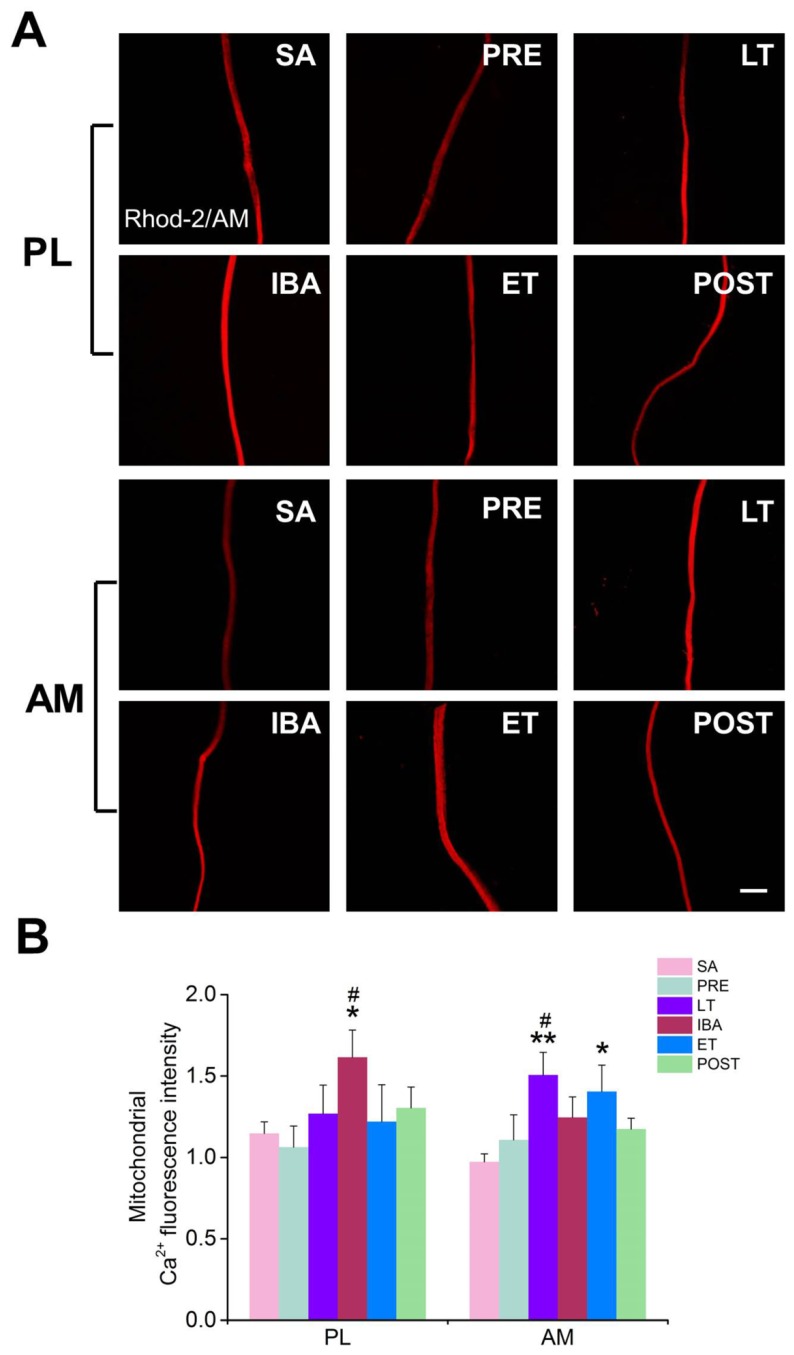
Changes in the mitochondrial Ca^2+^ concentration in PL and AM muscles during different periods. (**A**) Representative fluorescence images of single PL and AM muscle fibers. Scale bar = 100 μm. (**B**) Histogram depicting the mitochondrial Ca^2+^ fluorescence intensity in PL and AM muscles during different periods. PL, plantaris; AM, adductor magnus. SA, summer active group; PRE, pre-hibernation group; LT, late torpor group; IBA, inter-bout arousal group; ET, early torpor group; POST, post-hibernation group. Values are means ± SEM, n = 6–8. * *p* < 0.05 and ** *p* < 0.01 compared with SA; # *p* < 0.05 compared with PRE.

Compared with that in the SA or PRE groups, the mitochondrial Ca^2+^ level in the single muscle fibers was elevated to varying degrees during hibernation (in PL muscle of the IBA group and in AM muscle of the LT and ET groups) (Figure 5).

We comprehensively analyzed the changes in cytoplasmic, SR, and mitochondrial Ca^2+^ levels during different periods. Firstly, the opposite changes in cytoplasmic and SR Ca^2+^ suggest that SR Ca^2+^ participates in fluctuation of the cytoplasmic Ca^2+^ level during hibernation. In addition, the slight elevation in mitochondrial Ca^2+^ during hibernation may result from increased cytoplasmic Ca^2+^ or SR Ca^2+^ leakage. Further studies were subsequently carried out to explore the mechanisms involved in intracellular Ca^2+^ fluctuations during hibernation.

### 3.5. Relative mRNA and Protein Levels of Key Ca^2+^ Transport Proteins/Channels in Skeletal Muscle Fibers during Different Hibernation Periods

The mRNA and protein expression levels of several major Ca^2+^ transport proteins/channels located in the cytoplasm, SR, and mitochondria, including STIM1, ORAI1, RyR1, LETM1, PMCA3, SERCA1, MCU, MICU1, MICU2, and the free Ca^2+^ binding protein CALM, as well as the Pearson correlation coefficients for the co-localization of ORAI1 and STIM1, were detected to explore the potential mechanisms involved in intracellular Ca^2+^ fluctuation during hibernation. It should be clarified that, as mRNA expression is very sensitive to both internal and external environmental factors, large variability in the size of the error bars occurred in the mRNA statistical results. Therefore, we set a fold-change of ≥2-fold as the threshold for the biological significance of mRNA expression.

**Figure 6:**
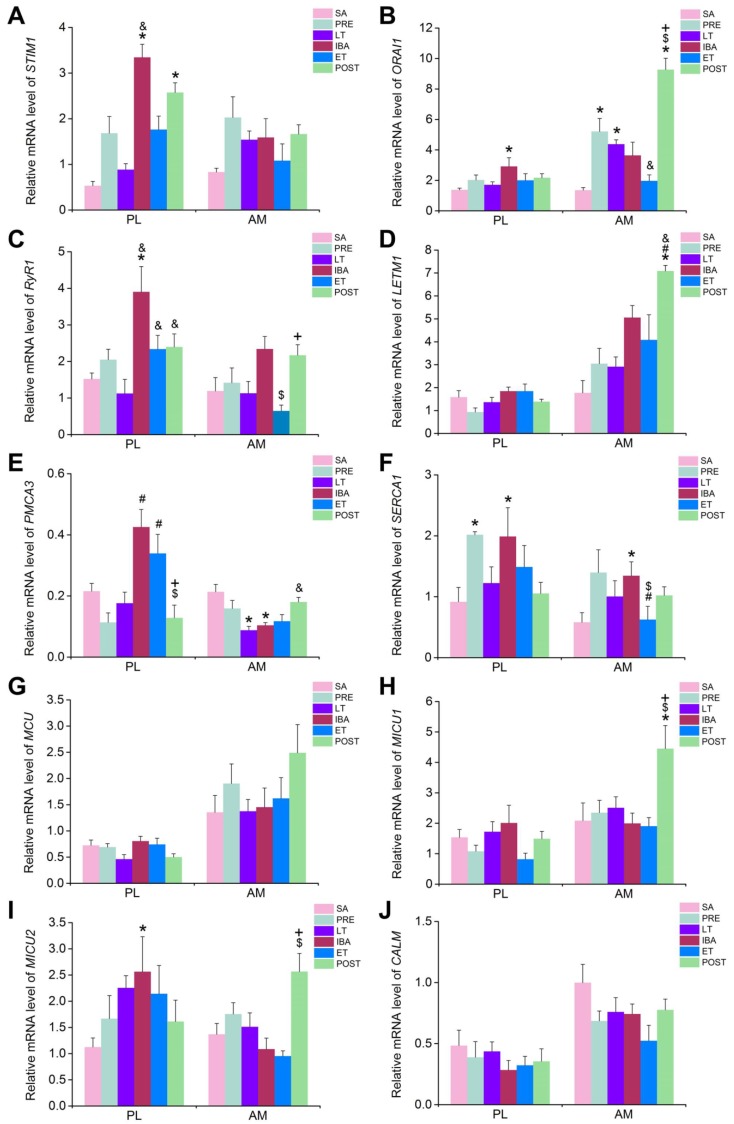
Changes in the mRNA expression of distinct Ca^2+^ transport and binding proteins in PL and AM muscles during different periods. Histograms depicting (**A**) stromal interaction molecule-1 (*STIM1*) mRNA expression, (**B**) *ORAI1* mRNA expression, (**C**) ryanodine receptor 1 (*RyR1*) mRNA expression, (**D**) leucine zipper-EF-hand containing transmembrane protein 1 (*LETM1*) mRNA expression, (**E**) plasma membrane Ca^2+^ ATPase (PMCA)3 mRNA expression, (**F**) SR Ca^2+^ ATPase 1 (*SERCA1*) mRNA expression, (**G**) mitochondrial calcium uniporter (*MCU*) mRNA expression, (**H**) mitochondrial calcium uptake 1 (*MICU1*) mRNA expression, (**I**) mitochondrial calcium uptake 2 (*MICU2*) mRNA expression, and (**J**) calmodulin (*CALM*) mRNA expression in PL and AM muscles during different periods. PL, plantaris; AM, adductor magnus. SA, summer active group; PRE, pre-hibernation group; LT, late torpor group; IBA, inter-bout arousal group; ET, early torpor group; POST, post-hibernation group. Values are means ± SEM, n = 6–8. * (*p* < 0.05 and fold change ≥ 2-fold), compared with SA; # (*p* < 0.05 and fold change ≥ 2-fold) compared with PRE; & (*p* < 0.05 and fold change ≥ 2-fold) compared with LT; $ (*p* < 0.05 and fold change ≥ 2-fold) compared with IBA; + (*p* < 0.05 and fold change ≥ 2-fold) compared with ET.

**Figure 7:**
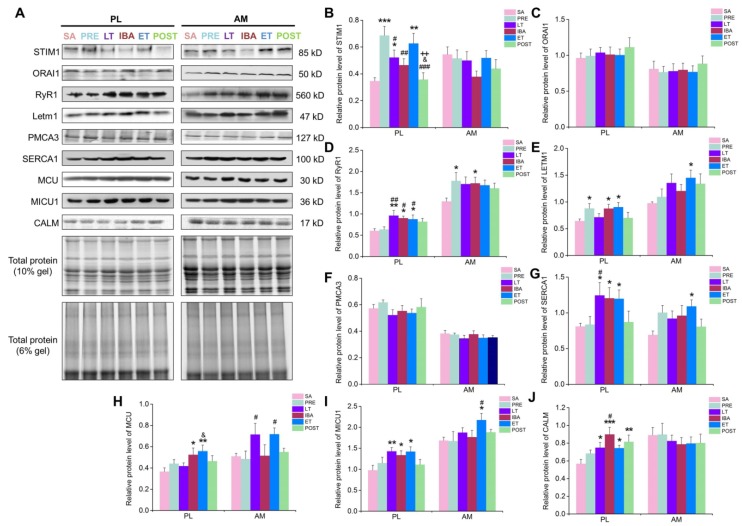
Changes in the protein expression of distinct Ca^2+^ transport and binding proteins in PL and AM muscles during different periods. Histograms depicting (**A**) representative Western blot images of STIM1, ORAI1, RyR1, LETM1, PMCA3, SERCA1, MCU, MICU1, and CALM in PL and AM muscles during different periods. Histograms depicting (**B**) STIM1 protein expression, (**C**) ORAI1 protein expression, (**D**) RyR1 protein expression, (**E**) LETM1 protein expression, (**F**) PMCA3 protein expression, (**G**) SERCA1 protein expression, (**H**) MCU protein expression, (**I**) MICU1 protein expression, and (**J**) CALM protein expression in PL and AM muscles during different periods. PL, plantaris; AM, adductor magnus. SA, summer active group; PRE, pre-hibernation group; LT, late torpor group; IBA, inter-bout arousal group; ET, early torpor group; POST, post-hibernation group. Values are means ± SEM, n = 6–8. * *p* < 0.05, ** *p* < 0.01, and *** *p* < 0.001 compared with SA; # *p* < 0.05, ## *p* < 0.01, and ### *p* < 0.001 compared with PRE; & *p* < 0.05 compared with LT; $ *p* < 0.05 and $$ *p* < 0.01 compared with IBA; ++ *p* < 0.01 compared with ET.

**Figure 8:**
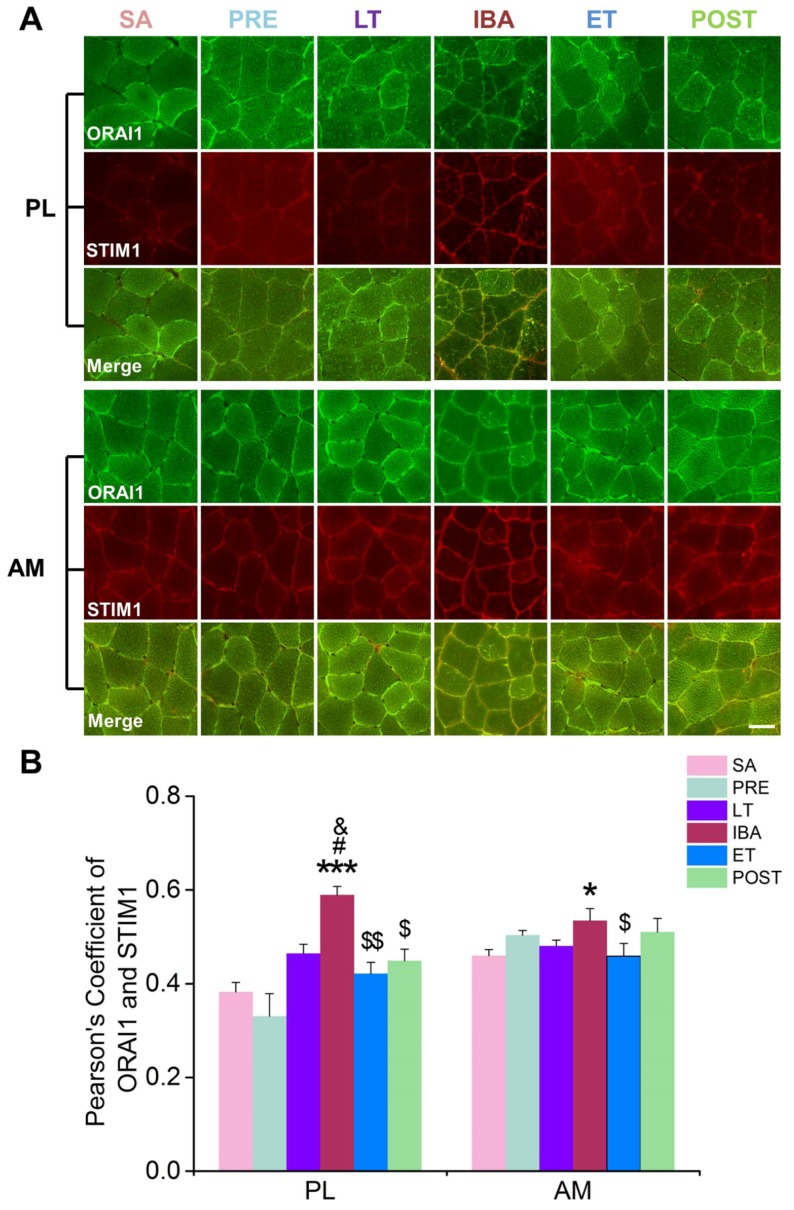
Changes in Pearson correlation coefficients of ORAI1/STIM1 in PL and AM muscles during different periods. (**A**) Representative fluorescence images in PL and AM muscles during different periods. 800× magnification, scale bar = 100 μm. (**B**) Histogram depicting Pearson correlation coefficients of ORAI1/STIM1 in PL and AM muscles during different periods. PL, plantaris; AM, adductor magnus. SA, summer active group; PRE, pre-hibernation group; LT, late torpor group; IBA, inter-bout arousal group; ET, early torpor group; POST, post-hibernation group. Values are means ± SEM, n = 6–8. * *p* < 0.05 and *** *p* < 0.001 compared with SA; # *p* < 0.05 compared with PRE; & *p* < 0.05 compared with LT; $ *p* < 0.05 and $$ *p* < 0.01 compared with IBA.

The mRNA and protein expression levels, as well as the Pearson correlation coefficients for the co-localization of ORAI1 and STIM1, were detected to explore the role of the SOCE channel in intracellular Ca^2+^ level fluctuation during hibernation. In the PL muscle, compared with that in the SA group, the mRNA expression levels of both STIM1 and ORAI1 increased during IBA (Figure 6A, B). Their protein expression levels showed an increasing (though non-significant) trend (Figure 7B, C). In addition, the Pearson correlation coefficients for the co-localization of ORAI1 and STIM1 were significantly elevated in the IBA group (Figure 8). In the AM muscle, the mRNA and protein expression levels of STIM1 and ORAI1 showed no significant change in the IBA group. However, Pearson’s correlation coefficients for the co-localization of ORAI1 and STIM1 exhibited slight increases in the IBA group. Overall, the Pearson’s correlation coefficients for the co-localization of ORAI1 and STIM1 in PL and AM muscle increased when ground squirrels aroused from torpor.

In view of the opposite changes between cytoplasmic and SR Ca^2+^ during hibernation, and to further explore whether the increased cytoplasmic Ca^2+^ during hibernation resulted from SR Ca^2+^ release, the mRNA and protein expression levels of RyR1 were measured. As shown in Figure 6C, in the PL muscle, compared with that in the SA group, the mRNA expression of RyR1 was significantly elevated in the IBA group. Its protein expression also increased (47–60%) in the distinct hibernation groups (Figure 7D). In the AM muscle, compared with that in the SA group, no significant change in RyR1 mRNA expression occurred (Figure 6C); however, its protein expression was significantly increased by 32% in the IBA group (Figure 7D). Overall, the protein expression of RyR1 in the PL and AM muscles increased significantly during hibernation (especially when animals aroused from torpor).

To further explore whether increased cytoplasmic Ca^2+^ during hibernation resulted from mitochondrial Ca^2+^ extrusion, the mRNA and protein expression levels of LETM1 were detected. In the PL muscle, compared with that in the PRE group, the mRNA expression of LETM1 increased significantly in the IBA and ET groups. Its protein expression level also significantly increased (37–40%) in the IBA and ET groups. In the AM muscle, compared with that in the SA group, the mRNA expression of LETM1 was dramatically elevated in the POST group (Figure 6D). The protein expression level was also elevated by (36–48%) in the LT, ET, and POST groups (Figure 7E). Overall, the mRNA and protein expression levels of LETM1 in the PL and AM muscles were elevated during hibernation and after post-hibernation.

The results showed that the cytoplasmic Ca^2+^ level partially recovered when the ground squirrels re-entered torpor after 12–24 h of IBA, which led us to consider the Ca^2+^ level recovery mechanisms involved in this process. The mRNA and protein expression levels of PMCA3 (a major plasma membrane Ca^2+^ ATPase in skeletal muscle) were first detected. The results showed that, compared with the SA and PRE groups, the mRNA level of PMCA3 in the PL muscle increased significantly in the IBA and ET groups (Figure 6E). A lower mRNA expression of PMCA3 was observed during hibernation in the AM muscle. In addition, the protein expression level of PMCA3 showed no significant differences among the different groups in either the PL or AM muscles (Figure 7F).

The mRNA and protein expression levels of SERCA1, a highly important Ca^2+^ pump located in the SR, were then detected. Compared with that in the SA group, the mRNA expression level of SERCA1 in the PL and AM muscles increased significantly in the IBA group (Figure 6F). Consistent with the changes in mRNA expression levels, the protein level of SERCA1 in the PL and AM muscles also increased significantly (43–58%) in the LT, IBA, and ET groups (Figure 7G). Overall, elevated SERCA1 mRNA and protein expression levels were observed in the PL and AM muscles during hibernation.

Mitochondria also plays a critical role in Ca^2+^ storage in skeletal muscle. Therefore, the mRNA and protein expression levels of the MCU complex, a major mitochondrial Ca^2+^ uptake channel, were determined. In the PL and AM muscles, compared with those in the SA group, no significant changes in mRNA expression levels of MCU, MICU1, or MICU2 were observed during hibernation (Figure 6G,H,I). However, the protein expression levels of MCU and MICU1 were increased significantly (29–51%) in the LT, IBA, and ET groups (Figure 7H,I).

In addition to the Ca^2+^ channels or pumps, free Ca^2+^ binding protein plays a major role in cytoplasmic Ca^2+^ levels. Therefore, we also detected the mRNA and protein expression levels of CALM. As shown in Figure 6J, in the PL and AM muscles, there were no significant differences in the mRNA expression levels of CALM among the different groups. However, the protein expression level was significantly increased during hibernation in the PL muscle, but showed no significant differences in the AM muscle (Figure 7J).

## 4. Discussion

**Figure 9:**
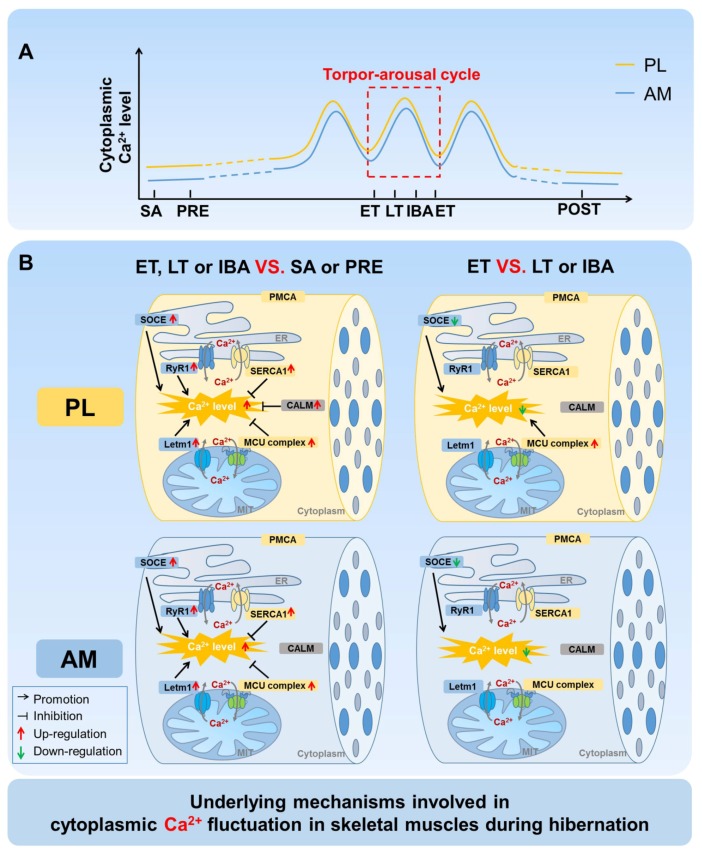
Graphical summary of the study. Our research focused on the potential roles of major Ca^2+^ channels and proteins in cytoplasmic Ca^2+^ fluctuations in skeletal muscles of Daurian ground squirrels during hibernation. (**A**) Fluctuation of cytoplasmic Ca^2+^ levels in skeletal muscle fibers during the torpor-arousal cycle. (**B**) Increased activation probability or protein expression of SOCE, RyR1, and LETM1 may participate in the periodic elevation of cytoplasmic Ca^2+^ during hibernation, whereas the increased expression of SERCA1, MCU, and CALM (only for PL) may be potential mechanisms through which hibernators can attenuate cytoplasmic Ca^2+^ and restore Ca^2+^ homeostasis during hibernation. Compared with that in LT or IBA groups, the decreased activation of SOCE and increased expression of MCU may participate in the partial down-regulation of cytoplasmic Ca^2+^. PL, plantaris; AM, adductor magnus. SA, summer active group; PRE, pre-hibernation group; LT, late torpor group; IBA, inter-bout arousal group; ET, early torpor group; POST, post-hibernation group.

A comprehensive time-course investigation was firstly carried out to measure the cytoplasmic, SR, and mitochondrial Ca^2+^ levels, as well as clarify the possible Ca^2+^ regulatory mechanisms, in the skeletal muscles of Daurian ground squirrels during different hibernation states. Fluctuations in intracellular Ca^2+^ levels were observed during the torpor-arousal cycle, with Ca^2+^ levels partially recovered when the animals aroused and then re-entered torpor (Figure 9A). Further investigation suggested that the Ca^2+^ proteins/channels and free Ca^2+^ binding protein located in the cytoplasm, SR, and mitochondria all participated in the fluctuation of intracellular Ca^2+^ (Figure 9B).

During prolonged hibernation, hibernators experience various stressful conditions, including prolonged inactivity, hypoxia, fasting, and repeated ischemia-reperfusion from the torpor-arousal cycle. Here, after several months of hindlimb inactivity during hibernation, slight decreases in muscle mass and single muscle fiber CSA (15–20%) were observed, suggesting that only limited skeletal muscle loss occurs in the PL and AM muscles, consistent with our previous report on PL and gastrocnemius muscles [36]. Obviously, hibernators can be considered good anti-atrophy models, and their unique skeletal muscle preservation mechanisms deserve further exploration. In view of the critical role of intracellular Ca^2+^ homeostasis in skeletal muscle maintenance, the present study focused on changes in Ca^2+^ levels in the different compartments (including cytoplasm, SR, and mitochondria) of skeletal muscle fibers during different hibernation stages. The results showed that the cytoplasmic Ca^2+^ levels in the PL and AM muscles were elevated to varying degrees during hibernation, suggesting that, similar to the phenomenon found in non-hibernators, prolonged skeletal muscle disuse during hibernation was accompanied by elevated cytoplasmic Ca^2+^ levels [37,38,39,40]. However, fluctuations in cytoplasmic Ca^2+^ levels were observed during the torpor-arousal cycle, with an increase during LT and partial recovery when the squirrels re-entered torpor after transient arousal. Therefore, periodic arousal from torpor may be a strategy employed by hibernators to adjust and stabilize cytoplasmic Ca^2+^ levels to effectively avoid excessive Ca^2+^-induced skeletal muscle loss or damage. Our previous study found that, compared with pre-hibernation levels, serum Ca^2+^ concentrations in ground squirrels increased significantly during hibernation and recovered after post-hibernation [41]. This raises the question of whether increased serum Ca^2+^ concentrations during hibernation influence cytoplasmic Ca^2+^ levels. SOCE is the main channel of extracellular Ca^2+^ influx. Higher co-localization coefficients of its two major components, i.e., STIM1 and ORAI1, represent a higher activation probability of SOCE [35]. Our results showed that the co-localization coefficients of STIM1 and ORAI1 increased significantly during LT and IBA, suggesting that the activation probability of SOCE increased during these two stages. Furthermore, the extracellular Ca^2+^ influx mediated by SOCE may be one reason for the increased cytoplasmic Ca^2+^ level during LT and IBA. In addition to extracellular Ca^2+^ influx, Ca^2+^ release from intracellular Ca^2+^ storage can also cause elevated cytoplasmic Ca^2+^ levels. RyR1 is the main Ca^2+^ release channel located in the SR. The results showed that its protein expression level was up-regulated to varying degrees during different periods of hibernation. Combined with the phenomenon that the Ca^2+^ concentration in the SR showed the opposite change to that of cytoplasmic Ca^2+^ during hibernation, the increased release of SR Ca^2+^ was likely a major reason for the increased Ca^2+^ level in skeletal muscles of ground squirrels during hibernation. In addition to SR Ca^2+^ release, several proteins also mediated Ca^2+^ efflux from the mitochondria to the cytoplasm. The present study showed that the protein expression of LETM1, a major channel that mediates Ca^2+^ efflux in the mitochondrial membrane, was increased to varying degrees during different periods of hibernation, intimating that Ca^2+^ release mediated by LETM1 in the mitochondria may also be involved in the increase in cytoplasmic Ca^2+^ levels during hibernation.

As a Ca^2+^ pump located in the cell membrane, PMCA3 can pump intracellular Ca^2+^ out of the cell. In the current study, however, the PMCA3 protein expression levels showed no significant differences during hibernation. Therefore, PMCA3 did not appear to play a substantial role in the fluctuation of cytoplasmic Ca^2+^ in hibernation. In contrast, the protein expression levels of SERCA1, a major Ca^2+^ uptake channel in the SR, were significantly up-regulated during different stages of hibernation, contrary to the decrease in SERCA activity found in non-hibernating animals under disuse conditions [42]. Our previous study showed that the protein content of SERCA2 in the soleus and extensor digitorum longus muscles of Daurian ground squirrels increased significantly during hibernation and IBA [7]. Other studies have also reported that SERCA activity is more resistant to temperature reduction in hibernating cardiac muscle than that in non-hibernating rats [43,44]. Such evidence indicates that, although faced with various stresses during hibernation, including a low temperature, low metabolism, and prolonged skeletal muscle disuse, SERCA1 still maintains high activity and can pump more cytoplasmic Ca^2+^ into the SR, thus avoiding an excessive increase in cytoplasmic Ca^2+^ and related skeletal muscle injury. In addition, the protein expression of MCU, a major Ca^2+^ absorption channel located in the mitochondrial membrane, was elevated during hibernation, and may therefore be another important mechanism for the absorption of Ca^2+^ into the mitochondria and alleviation of the Ca^2+^ level in the cytoplasm. However, we know that when the concentration of mitochondrial Ca^2+^ reaches a certain threshold, mitochondrial depolarization is triggered and, as a consequence, the pro-apoptotic protein Bax is activated, translocated, and inserted into the outer membrane via Bax/Bax-homo-oligomerization [45]. This is followed by the formation and opening of a mitochondrial permeability transition pore (mPTP), through which cytochrome C (a mitochondria-residing apoptogenic factor) is released into the cytosol, leading to the cleavage of nuclear DNA and cell apoptosis [3,46]. In the present study, the level of mitochondrial Ca^2+^ only increased slightly during hibernation. Therefore, the simultaneously higher expression of MCU and LETM1 may be a strategy employed by hibernating ground squirrels to control the balance between cytoplasmic and mitochondrial Ca^2+^, and thereby avoid mitochondrial Ca^2+^ concentration-induced skeletal muscle damage, such as cell apoptosis. As a free Ca^2+^ binding protein, CALM can relieve the elevation of cytoplasmic Ca^2+^ by binding to free Ca^2+^ in the cytoplasm. Our results showed that the protein expression of CALM in the PL muscle increased significantly, and the enhanced free Ca^2+^ binding capacity mediated by the elevated protein expression of CALM may thus be another important mechanism in hibernating ground squirrels to alleviate cytoplasmic Ca^2+^ levels during hibernation.

During the torpor-arousal cycle, the intracellular Ca^2+^ level showed increased-decreased fluctuation. Compared with that during LT and IBA, the cytoplasmic Ca^2+^ level in the skeletal muscles of the Daurian ground squirrels was down-regulated to varying degrees after re-entry into torpor (ET). Compared with levels in LT, the co-localization coefficients of STIM1 and ORAI1 decreased to varying degrees, representing decreased Ca^2+^ influx mediated by SOCE, which may be another critical mechanism used to avoid the intracellular Ca^2+^ increase caused by persistent Ca^2+^ influx during hibernation. In addition, we found that the higher protein expression of Ca^2+^ uptake channel MCU in ET may be another vital mechanism through which PL muscle can relieve cytoplasmic Ca^2+^ overload. It is worth noting that, after arousal from hibernation, the intracellular Ca^2+^ levels (i.e., cytoplasmic, SR, and mitochondrial) returned to the levels found in the SA and PRE groups, thus reflecting a strong and rapid Ca^2+^ recovery ability of these ground squirrels. We further found that the expression of Ca^2+^ transport proteins/channels or free Ca^2+^ binding protein also returned to the levels observed in the SA and PRE groups. This may be due to the sampling time of the POST group, which occurred 3 d after the squirrels aroused from torpor, and all physiological states had returned to normal.

By comparing and analyzing the mRNA and protein expression levels of each Ca^2+^ transporter protein, we found that the levels were not always consistent in the same stage. In particular, during LT, the mRNA levels of most Ca^2+^ transport proteins/channels were decreased or showed no significant change, whereas the corresponding protein expression levels were significantly increased. In view of these results, we checked the transcription and proteomic analysis results of the skeletal muscles during different hibernation stages (unpublished data) and found that the predicted results were consistent with our experimental findings. Previous studies have demonstrated that transcriptional elongation and initiation are essentially arrested during hibernation [47,48], which may be one of the main reasons for the lower mRNA expression of Ca^2+^ transport proteins/channels in skeletal muscles of Daurian ground squirrels during LT. In other words, the inconsistent changes in mRNA and protein expression levels may be the result of total inhibition at the transcriptional level during LT, although the corresponding protein expression was not affected during hibernation.

In summary, despite experiencing stresses such as a low temperature, low metabolism, and prolonged hindlimb inactivity during hibernation, hibernating ground squirrels still possess a strong Ca^2+^ operation ability. Here, by regulating the activity and protein expression of Ca^2+^ pumps, Ca^2+^ channels, and Ca^2+^ binding proteins in the cytoplasm, SR, and mitochondrial membrane, the dynamic balance of intracellular Ca^2+^ homeostasis was well-maintained during hibernation. Therefore, maintaining intracellular Ca^2+^ homeostasis and avoiding skeletal muscle injury caused by its disturbance appear to be priority strategies employed by hibernating squirrels to cope with the various stresses induced during the torpor-arousal cycle. 

## Figures and Tables

**Table 1:** Primers used for quantitative real-time PCR experiments.

Genes	Primer Sequence
***STIM1***	forward: 5′-CAGTTCTCATGGCCCGAGTT-3′
	reverse: 5′-GTGGGGAATGCGTGTGTTTC-3′
***ORAI1***	forward: 5′-CGCAAGCTCTACTTGAGCCG-3′
	reverse: 5′-CATCGCTACCATGGCGAAGC-3′
***RYR1***	forward: 5′-GGTACTGGTCGGGATACCCT-3′
	reverse: 5′- GACCTCGGGACTCTCAATCA-3′
***Letm1***	forward: 5′-ACTGGTCCCTTTCCTGGTCT-3′
	reverse: 5′-CTTCAGCCTCTCCTCCTTGA-3′
***PMCA3***	forward: 5′-CGGCGGTCTTCGGTCCTCAG-3′
	reverse: 5′-TGGGCTTGGCGGCAGAGAG-3′
***SERCA1***	forward: 5′-GGTACTGGTCGGGATACCCT-3′
	reverse: 5′-GCTGGATAGAGCCTGTGACC-3′
***MCU***	forward: 5′-TGGTGTGTTTTTACGGCAAC-3′
	reverse: 5′-TCATCAAGGAGGAGGAGGTC-3′
***MICU1***	forward: 5′-TGGGTATGCGTCACAGAGAT-3′
	reverse: 5′-GATGGTCAGTTTCCCCTTGA-3′
***MICU2***	forward: 5′-TGACACCACGAGACTTCCTCT-3′
	reverse: 5′-GATTCCTGCCAATACCTCCTC-3′
***CALM***	forward: 5′-GGCACCATTGACTTCCCAGA-3′
	reverse: 5′-TCTGCCGCACTGATGTAACC-3′
***α-tubulin***	forward: 5′-AATGCCTGCTGGGAGCTCTA-3′
	reverse: 5′-CAGCGCCTGTCTCACTGAAG-3′

## Supplementary Materials

The following are available online at https://www.mdpi.com/2073-4409/9/1/42/s1, Figure S1: Represent image of the complete SDS-PAGE lane for each antibody used in present study.
