# Supplementary material for: Priority Strategy of Intracellular Ca2+ Homeostasis in Skeletal Muscle Fibers during the Multiple Stresses of Hibernation
Source: Cells. 2019 Dec 22;9(1):42. doi: 10.3390/cells9010042 (PMC7016685; doi:10.3390/cells9010042)
Supplement: Supplementary file 1 [file cells-09-00042-s001.pdf]

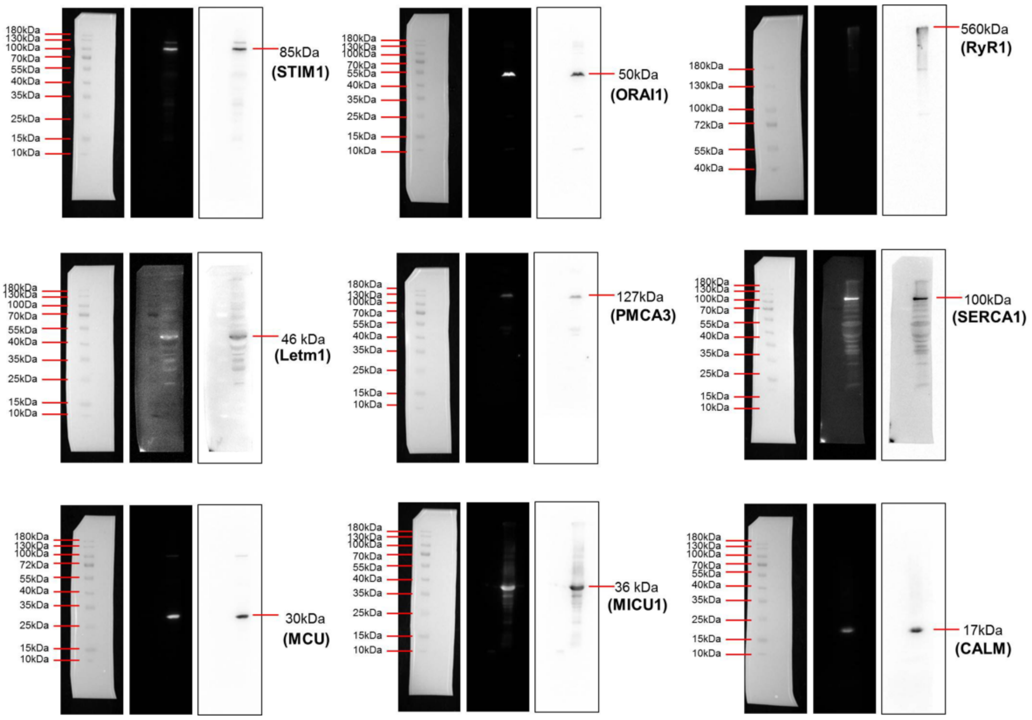

Figure S1 Represent image of the complete SDS-PAGE lane for each antibody used in present study. The left image shows the PVDF immunoblotting membrane under white light condition captured by the electrophoresis gel imaging analysis system, the mediate image shows the protein bane under no light condition, and the right image shows the reverse colored image of the mediate image. It is obvious that, all antibodies used in present study shows good specificity for the protein of interest, which can detect the target bands near the corresponding molecular weight.
